# When Multidrug-Resistant Organism (MDRO)-Positive ICU Patient Isolation and Cohorting Is Not Feasible, What Comes Next?

**DOI:** 10.7759/cureus.13636

**Published:** 2021-03-01

**Authors:** Stelios Iordanou, Lakis Palazis, Chrystalla Timiliotou-Matsentidou, Michalis Mendris, Vasilios Raftopoulos

**Affiliations:** 1 Intensive Care Unit, Limassol General Hospital, Limassol, CYP; 2 Intensive Care Unit, Nicosia General Hospital, Nicosia, CYP; 3 Microbiology Department, Limassol General Hospital, Limassol, CYP; 4 HIV Surveillance Division, Hellenic National Public Health Organization, Athens, GRC

**Keywords:** horizontal infection, mrsa, mdro, contact precautions, icu, isolation, cohorting

## Abstract

Background

The need for the implementation of an infection prevention strategy that included patient isolation and a cohorting procedure emerged in our ICU. Yet, isolation, as well as cohorting, were not feasible due to certain barriers associated with a high colonization pressure, open-plan ICU, inadequate bed separation, a limited number of isolation rooms, and nursing shortage. Despite these limitations, we tried to upgrade our ICU’s infection prevention efforts by developing the "universal use of contact precautions approach" for infection prevention and control for all the patients with and without multidrug-resistant organisms (MDROs), cohorting, and single room isolation. The study aimed to evaluate the effectiveness of our approach.

Methods

A prospective cohort study using surveillance screening cultures for *Methicillin-resistant Staphylococcus aureus* (*MRSA*) and MDROs for a period of 18 weeks from October 1, 2018, to January 31, 2019. The main purpose of the approach was to isolate all patients (regardless of their MDRO/MRSA status) in their own bed space as if they were in an isolation room for the entire duration of their ICU hospitalization, in such a way as to prevent horizontal transmission of infection (infection acquisition) in our open-plan ICU.

Results

Seventy-eight patients were admitted to our ICU for a total of 942 patient-days; a total of 432 swabs were collected during the study period. A total of 17 (21.8%) patients were admitted with a pre-existing infection while two (2.5%) patients acquired an infection during their ICU stay (one with *Acinetobacter baumannii *andone* *with* Pseudomonas aeruginosa*; 1.28 acquisition per 1000 patient-days). No transmission was documented for *Klebsiella pneumoniae*, *Enterococcus faecalis,* and *Staphylococcus aureus.*

Conclusions

Our MDRO acquisition rates suggested that the implementation of our infection control strategy potentially prevents the horizontal transmission of pathogens in an open-plan ICU, despite the high colonization pressure and the lack of isolation and cohorting procedures.

## Introduction

Healthcare-associated infections (HAIs) are a significant cause of adverse healthcare-related events in hospitalized patients. Despite the relatively low percentage (8% to 15%) of hospital admissions, ICU patients account for 40% to 50% of all hospital infections [1]. Infection events are a significant factor that contributes to the increased cost of care [2-3] increased mortality and morbidity, as well as poor patient outcomes. This is attributed to the greater severity of ICU patients’ conditions, the increased use of invasive devices in the ICU, and the underlying high-risk ICU environment. 

These infections can be transmitted easily to other patients and/or the environment by direct and indirect contact. Direct contact occurs whenever pathogens are transferred from one infected to another without a contaminated intermediate object or person being involved (such as from the caregiver to the patient or vice versa). Indirect contact includes the transfer of an infectious agent by means of a contaminated object or third person [4]. Infection transmission routes must be blocked to prevent the horizontal transmission of microorganisms. The risk of transmission is minimized by using patient isolation and cohorting procedures as advised in the published literature [5]. Such a procedure requires the combination of isolation and contact precaution measures as well as grouping patients.

According to the US Centre for Disease Control and Prevention (US CDC), additional consideration should be given to patients with multiple drug-resistant organisms (MDROs) in ICUs [6] using measures that include single room isolation and dedicated specific staff to provide the bulk of the patient’s care rather than all staff available.

In case single rooms are not available to isolate MDRO-positive patients, the use of cohorting is highly advised, which is translated into grouping these patients with others who have cultured positive with the same organism(s) [5]. Yet isolation, as well as cohorting, was not feasible at that time due to the design of our ICU. Consequently, we were unable to comply with the relevant recommendations in the published literature.

Despite these limitations, we tried to upgrade our efforts with regard to MDROs spreading across ICU patients by developing and implementing a new sustainable approach.

The aim of the study was to assess the effectiveness of the aforementioned approach to prevent MDROs from spreading across ICU patients.

## Materials and methods

Study design and data collection

A prospective cohort, active MDROs screening surveillance study was conducted using an ICU-based protocol (European Centre for Disease Prevention and Control (ECDC) Healthcare-Associated Infections (HAI)-ICU Protocol, v1. 01 standard edition) for a period of four months from October 1, 2018, to January 31, 2019. The standard ECDC HAI-ICU protocol was enhanced by the addition of screening to the admission section with reference to MDRO documentation and their resistance pattern in such a way as to increase the effectiveness of infection surveillance. This is described in detail below.

All patients admitted in the ICU were screened for methicillin-resistant *Staphylococcus aureus* (MRSA) and MDROs (MDROs [rectal sample] and MRSA [nasal sample]) by obtaining nasal and rectal culture swabs during admission, followed by weekly screening and on discharge.

In the event of MDRO-positive patients, pathogen/s species and resistance pattern were linked with the rest of the infection positive ICU patients (colonization pressure), in order to detect the patient/infection source.

Standard laboratory methods were used to identify microorganisms using automated methods - Phoenix 100 (Becton, Dickinson and Company, Franklin Lakes, New Jersey) and Vitek II (bioMérieux, Marcy-l'Étoile, France).

Statistical analysis

All analysis was performed using R version 3. 5. 1 (R Foundation for Statistical Computing, Vienna, Austria), while 95% Poisson confidence intervals for the incident density of colonization were calculated by using the package exacti (exacti.us. Brooklyn, NY). Graphs of colonization pressure and incident density were created using the package ggplot2 (Tidyverse, R Studio).

Setting

This study was conducted in the ICU of a major, public, secondary general referral hospital with a total bed capacity of 400 beds and 28,000 hospital admissions per year. The unit is a closed-type adult ICU of open plan design, case mixed, with eight beds capacity. Primarily, it serves the south of the island; however, patients may be admitted from the private sector and other public hospitals throughout the Republic of Cyprus.

Our ICU is one of the two closed-type ICUs in the public hospitals across the island. Prior to the coronavirus disease 2019 (COVID-19) pandemic, a total number of 25 closed-type and 27 open-type ICU beds operated. A total of 65 ICU beds are offered in private hospitals across the island. Two more closed-type ICUs have been established recently in the biggest hospital on the island (a total bed capacity of 600 beds) due to the need for COVID-19 patients management.

Problematic region

The lack of patients’ bed floor area and the limited space between beds are the two major problems associated with the daily operation of the ICU. Our patient bed floor area is 14.4 m^2^ (3.6 m * 4 m), the bed to bed space is two meters or less with an aisle 1.8 m wide immediately adjacent while the minimum international standards recommend at least 20 m^2^ floor area for each bed and 2.5 m for an open aisle [7]. There is no physical barrier between patient’s bed spaces while the one and only single-bedded isolation room is used primarily for protective isolation of the immunocompromised patients but not exclusively.

New approach: “the universal use of contact precautions”

The main purpose of the approach was to isolate all patients (regardless of their MDRO/MRSA status), in their own bed space, as if they were in an isolation room for the entire duration of their ICU stay in such a way as to prevent the horizontal transmission of infection (infection acquisition) in our open-plan ICU. 

**Figure 1:**
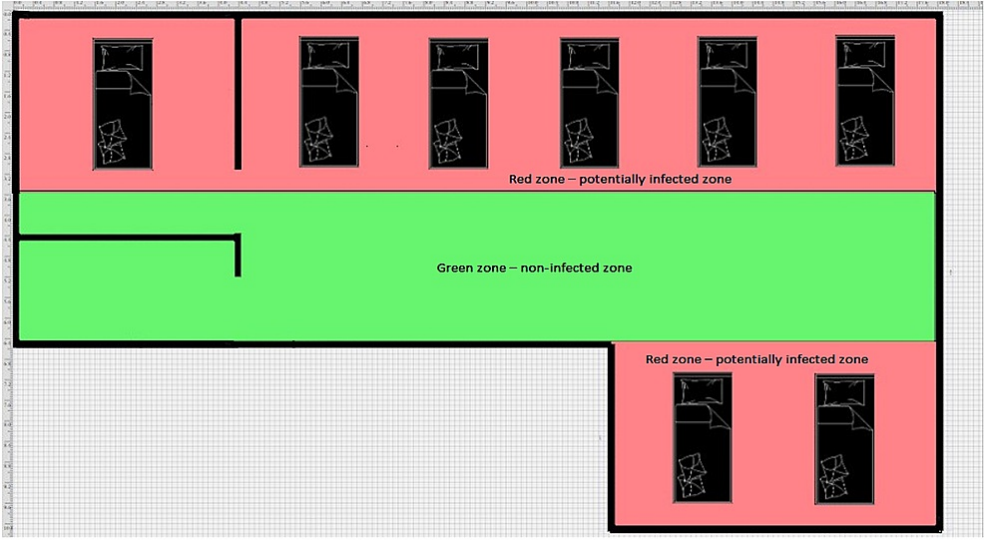
Red and green grading zones

Since isolation rooms and cohorting MDRO-positive patients were not feasible, we divided the open-plan ICU space virtually (rather than physically) into two grading zones (red and green zone) (Figure 1) followed by behavioral restrictions to be applied, along with contact precautions, to all patients from their admission to the discharge or death (universal use of contact precautions) based on these zones.

In addition, visitor surveillance by a nurse in charge was introduced to ensure that visitors follow our infection prevention recommendations. The nurse in charge was responsible for monitoring not only a specific patient at all times but also all the professionals that came in contact with the patients and their surrounding space (physicians, physiotherapists, nurses, cleaning and nursing assistants, as well as other visitors’ professionals).

Red Zone: Potentially Infected Zone

The “red zone” was defined as the floor area around the patient’s bed together with everything it contained. This was considered a potentially infected zone. The patient, bed, trolleys, monitors, ventilators, infusion pumps, and other necessary equipment for the patient's treatment and care were located in the red zone. In order to assure the isolation of the patients in their own space, each patient had his/her own red zone and contact precautions and behavioral restrictions applied accordingly.

Contact precautions for the red zone: Entering a red zone required contact precautions. Specifically:

- In case of potentially significant contact with the patient, contact precautions were employed (gloves, isolation gown, etc. (depending on the procedure to be performed)).

- In case of potentially significant contact with MDRO-positive or suspected positive patient, with airborne pathogens as well as by respiratory secretions, a face mask was worn by the staff.

- In case that contact with the patient was not considered significant (e.g., changing a propofol vial or silencing a monitor alarm, switching off an infusion pump, etc.), only gloves were used.

Before exiting the red zone, all used and disposable equipment was disposed of in the contaminated waste bins located within the specific zone (gloves, face masks, isolation gown, and any other used disposable equipment (syringes, gauzes, etc.)). Non-disposable equipment was cleaned with an appropriate disinfectant before leaving the patient’s specific red zone.

Behavioral restrictions in the red zone: There was a one-way transfer of disposables or other equipment into a red zone; passing disposable or other equipment between patient’s red zones was forbidden; and hand hygiene was performed immediately before entering and after exiting a red zone.

Green Zone: Non-Infected Zone

The “green zone” was considered to be the non-infected zone, as this was considered the rest of the ICU area with the exception of the red zones. In the green zone, all staff and visitors were allowed to have contact with the equipment and the environment without any precautions. Equipment such as ultrasound machines, endoscopy equipment, etc. was stored in that zone.

Contact restrictions in the green zone: None, apart from contact with the floor.

Behavioral restrictions in the green zone: Daily routines such as hand hygiene and use of gloves when dealing with biological substances; safe disposal of biomedical waste, even non-infected.

## Results

Seventy-eight patients have been admitted (78), and a total of 432 swabs were collected for the detection of MRSA and MDROs, during the study period. A total of 17 (21.8%) patients were admitted with an infection while two (2.5%) patients acquired an infection during their ICU stay. Patients were monitored for a total of 942 ICU days leading to MDROs' incident density of colonization on the admission of 17 per 1000 days 95% CI, 12.1 - 31.4]. Furthermore, the overall colonization pressure was 19/78 (24.4%) during the study period.

The overall admission colonization pressure of *Acinetobacter baumannii *(ACIBAU) was 7.7%, for *Pseudomonas aeruginosa* (PSAER) 6.4%, for *Enterococcus faecalis* (ENCFAE) 6.4%, for *Staphylococcus aureus* (STAAUR) 6.4%, and for *Klebsiella pneumoniae* (KLEPNE) 3.84%. The number of MDRO-positive patients, the colonization pressure, incident density across each type of infection, and weekly colonization pressure are depicted in Table 1 and Table 2.

**Table 1:** Number of infected patients (N = 78) †Some of the patients admitted with more than one MDRO ACIBAU: *Acinetobacter baumannii*; PSAER: *Pseudomonas aeruginosa*; ENCFAE: *Enterococcus faecalis*; STAAUR: *Staphylococcus aureus*; KLEPNE: *Klebsiella pneumoniae*; MDRO: multiple drug-resistant organism

Infection	On Admission	During ICU Stay	Colonisation Pressure Upon Admission	ICU Acquisition per 1000 days (%)	Incident Density of Colonisation (95% CI) per 1000 Days
ACIBAU	6 (35.3%)	1 (50%)	6/78 (7.7%)	1.28 (1.26)	7.4 (2.9 – 15.3)
PSAER	5 (29.4%)	1 (50%)	5/78 (6.4%)	1.28 (1.26)	7.4 (1.7 – 12.4)
ENCFAE	5 (29.4%)	0 (0%)	5/78 (6.4%)	0 (0%)	5.3 (1.7 – 12.4)
STAAUR	5 (29.4%)	0 (0%)	5/78 (6.4%)	0 (0%)	5.3 (1.7 – 12.4)
KLEPNE	3 (17.6%)	0 (0%)	3/78 (3.8%)	0 (0%)	3.18 (1.1 – 10.8)
Total Patients	17† (100%)	2 (100%)			

**Table 2:** Weekly colonization pressure by infection per week ACIBAU: *Acinetobacter baumannii*; PSAER: *Pseudomonas aeruginosa*; ENCFAE: *Enterococcus faecalis*; STAAUR: *Staphylococcus aureus*; KLEPNE: *Klebsiella pneumoniae*

Study Week	Calendar Week	PSAER	KLEPNE	ACIBAU	ENCFAE	STAAUR	Total Patients^†^
1	40	1 (16.7%)	1 (16.7%)	1 (16.7%)	0 (0.0%)	0 (0.0%)	6
2	41	1 (9.1%)	1 (9.1%)	1 (9.1%)	0 (0.0%)	0 (0.0%)	11
3	42	1 (10.0%)	1 (10.0%)	1 (10.0%)	1 (10.0%)	0 (0.0%)	10
4	43	2 (16.7%)	2 (16.7%)	1 (8.3%)	1 (8.3%)	0 (0.0%)	12
5	44	2 (15.4%)	2 (15.4%)	2 (15.4%)	1 (7.7%)	0 (0.0%)	13
6	45	2 (13.3%)	2 (13.3%)	2 (13.3%)	1 (6.7%)	0 (0.0%)	15
7	46	2 (16.7%)	1 (8.3%)	2 (16.7%)	2 (16.7%)	0 (0.0%)	12
8	47	2 (20.0%)	1 (10.0%)	1 (10.0%)	2 (20.0%)	0 (0.0%)	10
9	48	1 (12.5%)	1 (12.5%)	0 (0.0%)	2 (25.0%)	0 (0.0%)	8
10	49	1 (12.5%)	1 (12.5%)	0 (0.0%)	2 (25.0%)	0 (0.0%)	8
11	50	2 (20.0%)	1 (10.0%)	0 (0.0%)	1 (10.0%)	0 (0.0%)	10
12	51	2 (16.7%)	1 (8.3%)	1 (8.3%)	1 (8.3%)	0 (0.0%)	12
13	52	2 (18.2%)	1 (9.1%)	1 (9.1%)	1 (9.1%)	0 (0.0%)	11
14	1	1 (7.7%)	1 (7.7%)	2 (15.4%)	1 (7.7%)	0 (0.0%)	13
15	2	1 (7.1%)	1 (7.1%)	1 (7.1%)	0 (0.0%)	2 (14.3%)	14
16	3	2 (11.8%)	1 (5.9%)	0 (0.0%)	0 (0.0%)	3 (17.6%)	17
17	4	2 (20.0%)	1 (10.0%)	0 (0.0%)	0 (0.0%)	3 (30.0%)	10
18	5	2 (25.0%)	1 (12.5%)	0 (0.0%)	0 (0.0%)	3 (37.5%)	8

**Figure 2:**
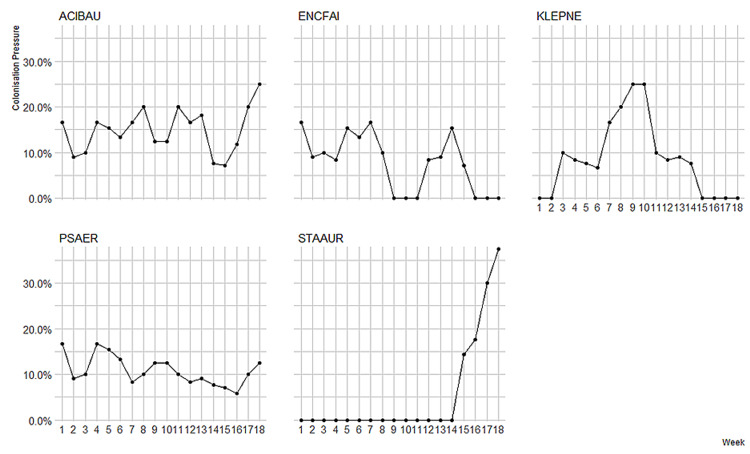
Colonization pressure over the 18-week study period

**Figure 3:**
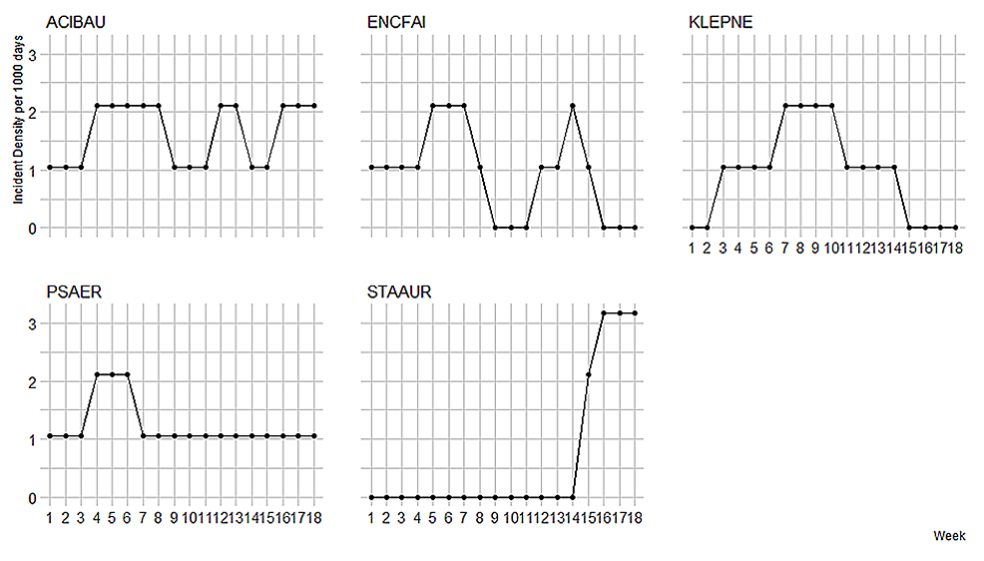
Incidence density of colonization over the 18-week study period

Figure 2 presents the weekly colonization pressure per MDRO per calendar week during the study period; Figure 3 presents the incidence density rates for MDROs per calendar week during the study period.

## Discussion

All patients admitted to the ICU are potentially symptomatic or asymptomatic MDROs and/or MRSA carriers despite the department they were admitted or transferred from. Their MDRO/MRSA status is usually detected three to four days postadmission after the initial surveillance culture results become available.

According to the US Center for Diseases Control and Prevention (CDC) and the Hospital Infection Control Practices Advisory Committee (HICPAC) in their updated 2007 guidelines, standard precautions (SP) should be used in all cases while the use of gloves, gowns, and eye protection is recommended in cases of high-risk exposure to blood or body secretions. Contact precautions are also recommended for patients colonized or infected with MDROs followed by a high risk of cross-transmission such as MRSA or *vancomycin-resistant enterococci* (VRE). Further, in the case of MDRO positive patients, isolation is recommended or the use of some form of cohorting procedure whenever this is not feasible [8].

Therefore, there is a gap between admission and the availability of the first culture result, which constitutes a “grey zone” with regard to the use of contact precautions or not. This gap in the recommendations, for instance, increases the risk for asymptomatic patients admitted to an ICU colonized with MDROs to be the potential carriers or recipients of horizontally transmitted potentially life-threatening infections, especially in open-plan ICU.

Having that “recommendation gap” in mind, we nursed all patients on admission as if they were potentially MDRO carriers and extended the use of contact precautions (universal use of contact precautions) to all the patients from their admission to the discharge or death. Contact precautions are time-consuming, but no statistically significant correlation was observed in the relevant studies [9] between any resultant delays and adverse events such as pressure ulcers, falls, and errors in medication administration. However, in order to minimize potential delays within our ICU, necessary protective equipment was placed in the green zone, close to each of the patients and their red zone(s). The universal-use of contact precautions replaced the “recommendations gap.” However, difficulties still existed with regard to isolation and cohorting MDRO-positive patients. 

The use of isolation and cohorting procedures [5] is a critical issue, as it has resulted in the successful control of outbreaks (gram-positive and negative organisms (VRE or MRSA extended-spectrum beta-lactamase (ESBL)-producing *Klebsiella pneumoniae *and MDR* Serratia marcescens*)) in several studies [10-13]. Yet, the implementation of the procedures requires an appropriate ICU design and an appropriate nurse-to-patient ratio [14].

In our ICU, the use of isolation or cohorting for MDRO-positive patients was not feasible due to the lack of room space. Consequently, the development of the new approach combined contact precautions for all the patients as previously described, and ICU grading zones aiming to isolate the patients in their own space for the entirety of their ICU hospitalization and without unfeasible physical (single room) isolation and cohorting. A multicentred study [15] supported that using contact precautions in multiple-bed rooms may have the same effect on the prevention of ESBL-producing *Enterobacteriaceae*, as single-room isolation. Nevertheless, the study included non-ICU patients and only ESBL-producing *Enterobacteriaceae*.

While patient isolation is considered to be a safe practice, it should be noted that the onset of adverse events that involve isolated and non-isolated patients can suggest otherwise. According to a previous study, isolated patients were twice as likely to experience an adverse event during hospitalization (31 versus 15 adverse events per 1000 days) and seven times more likely to experience a preventable adverse event (20 versus three adverse events per 1000 days) [16]. However, these results cannot be extended to our ICU since our patients were hospitalized in a high-visibility, open-plan ICU, in which adverse unobserved events were less likely to occur.

Our approach was applied to patients’ visitors given that they interact significantly with their patients during the visiting hours even though no published literature reported any link between visitors and MDRO spread. On the contrary, an association was found between patient's visitors/relatives and tuberculosis outbreaks in 1986 [17] and 1995 [18], plus severe acute respiratory syndrome (SARS) in 2003 [19] and two further cases in 2004 [20-21]. In any case, our prevention efforts were applied to all patients’ visitors considered to be potential MDRO carriers due to close contact with the ICU-hospitalized patients during the visiting hours. In order to maximize the patients’ visitors' compliance with our infection prevention plan, a visitors’ surveillance nurse role was introduced for monitoring and advising visitors to maintain this plan.

A significant reduction in MDRO acquisition was noted in ICU patients after the implementation of our approach. Yet, the actual number, proportion, and rates of reduction were not known due to the lack of a surveillance system.

The effectiveness of our universal use of contact precautions approach relies on the avoidance of MDRO acquisition due to ICU stay, but it should be considered together with the ICU’s colonization pressure since this is strongly associated with an increased risk of MDRO acquisition [22]. According to a Greek study, 5.6% of all patients admitted to an ICU were already colonized with ACIBAU spp. on admission, whereas 15.7% have been infected during their stay. They concluded that high levels of colonization pressure and the admission of more than two carriers per week independently increased the infection acquisition risk.

During the study period, MDRO acquisition in our ICU was confirmed in two patients (2. 5% n=78): a case of PSAER and a case of ACIBAU. These patients were negative to these organisms in the weeks before their positive cultures on the ICU, their MDRO-positive culture results (resistance pattern) were correlated with the rest of the MDRO-positive patients; thus the source of the patients’ infections was confirmed. 

In our study, 7.7% (6, n=78) of all patients admitted were colonized with ACIBAU and HAI accounted for 1.26% (one patient, n=78) during their ICU stay. ACIBAU proportion on admission is higher than the Greek study (5. 6%) [23], yet ACIBAU acquisition in our ICU is significantly less (1.26 versus 15.7%, [1.28 per 1000PD]). This is a significant finding, as the aforementioned study was the first that explored the association between ACIBAU colonization pressure and the risk of acquisition, although this cannot extend to our study since the association is not a positive one. Thus, our findings suggested that the infection control approach could be considered effective in preventing the horizontal spread of ACIBAU, despite the high proportion of ACIBAU-positive patients on the admission.

In our sample, 6.4% was found to be positive to PSAER on the admission, a rate that is lower than that reported in the US (11.6%) [24], France (17%) [25], and the Netherlands (9.2%) [26]. Despite our low infection rates compared with other studies, one of our patients acquired PSAER during the ICU stay, resulting in an ICU acquisition proportion of 1.26 (1.28 per 1000PD). Our rate is lower compared to those reported in the studies mentioned previously (6.4% versus 26% & 16%) [25,27]; our study reported that infection or colonization rates on admission were half of those reported in the US and one third those reported in French studies, yet our in-hospital acquisition results are 12 to 20 times less. Consequently, we consider that our infection prevention approach is effective in avoiding PSAER horizontal spread in the ICU environment.

PSAER and ACIBAU (one acquisition, respectively) were the only organisms documented as ICU-acquired, during the study period. Six-point four percent (5/78) were carriers of MRSA on admission, a proportion that is higher than that reported in a multicenter Chinese study (3.4%) [28], similar to that in French ICUs (4.2%-6.9%) [29]. Despite the high prevalence of MRSA in our ICU, no horizontal MRSA transmission was documented in our sample (n=78). No MRSA decolonization practices had been introduced during the study period that might interfere with our results.

Positive to KLEPNE carriers accounted for 3.8% (3/78) of all patients admitted in our ICU with no acquisition being documented. This is important given that KLEPNE can cause outbreaks due to its ability to spread rapidly within healthcare facilities mainly by the hands of the hospital personnel, that is, amongst others, a significant KLEPNE reservoir [30]. ENCFAE positive cultures were found on five (6.4%) of all of our ICU patients admitted during the study period, without any ICU acquisition being documented.

Overall colonization pressure was 24.4%, meaning that 24.4% of our patients were carriers of at least one MDRO on ICU admission and ICU stay. Therefore our colonization pressure was nearly four times higher compared to the rate in the Greek study (5.6%) [23], higher compared to the French study (13%), but lower compared to a multicenter Chinese study (36%) [28]. It is noted that in the French study, although MDRO acquisition is reported, colonization pressure data are not. The overall colonization pressure of ACIBAU was 9%, MRSA 6.4%, KLEPNE 3.84%, ENTFAE 6.4%, and PSAER 7.7% during the study period (admission rates plus ICU acquisitions).

The acquisition rates for each of the MDROs in our ICU were as follows: 1.28 cases of ACIBAU, 1.28 cases of PSAER per 1000 PD, respectively, and zero for KLEPNE, VRE, and MRSA per 1000 PD. 

When it comes to the comparison of the aggregated HAI acquisition rates during ICU hospitalization, our overall rates (2.56 per 1000 PD), were much lower than those in other published studies. The acquisition rate in the Greek study was 15.7 [23], in the Netherlands, it was 14 [26], and in a Chinese study, it was 35 per 1000 PD [28].

## Conclusions

Isolation and cohorting strategies may be beneficial, although they cannot be implemented in all cases; especially in open-plan ICUs. The universal use of contact precautions approach potentially prevents the horizontal transmission of pathogens in a high disease severity and high colonization pressure ICU. The MDRO acquisition rates in our ICU suggest that the implementation of our infection control strategy potentially prevents the horizontal transmission of pathogens in the ICU, despite high colonization pressure and lack of isolation and cohorting procedures for MDROs.

We strongly believe that the universal use of contact precautions approach should be considered as an approach for enhancing the prevention and control of HAIs (MDRO or not) in the ICU either as a sole prevention intervention or whenever MDRO-positive patients' isolation and cohorting have failed to be implemented.
